# Interplay Between Virulence Genes, Antimicrobial Resistance Profiles, and Biofilm Formation in *Klebsiella pneumoniae* Causing Urinary Tract Infections in North Lebanon

**DOI:** 10.1002/mbo3.70336

**Published:** 2026-06-17

**Authors:** Mazen Zaylaa, Rayan Farha, Jana Taleb, Amal Saad, Omar El Gharbawy, Ricardo Sarraf, Ibtihal Kassam, Chadi Hamdach, Nisrine Bissar

**Affiliations:** ^1^ Faculty of Medicine Beirut Arab University Beirut Lebanon; ^2^ Department of Medical Laboratory Technology, Faculty of Health Sciences Beirut Arab University Beirut Lebanon; ^3^ Department of Laboratory Medicine Monla Hospital Tripoli Lebanon; ^4^ Department of Laboratory Medicine Dar Al Shifa Hospital Tripoli Lebanon; ^5^ Department of Microbiology Kadisha Medical Laboratories Tripoli Lebanon

**Keywords:** antimicrobial resistance, biofilm formation, Klebsiella pneumoniae, putative hypervirulent strain, urinary tract infection, virulence genes

## Abstract

*Klebsiella pneumoniae* (*K. pneumoniae*) is a major cause of urinary tract infections (UTIs) and poses a growing public health concern due to multidrug resistance and virulence potential. This study aimed to characterize antimicrobial resistance, biofilm formation, and virulence gene distribution among urinary *K. pneumoniae* isolates from North Lebanon and to explore correlations between these factors. A total of 153 non‐duplicate isolates from hospital and community settings were analyzed for antibiotic susceptibility, hypermucoviscosity, biofilm formation, and presence of key virulence (fimH, mrkD, magA, rmpA, entB, iucA, iroN, kfu) and β‐lactamase (blaTEM, blaSHV, blaCTX‐M) genes. Adhesion‐associated genes fimH and mrkD were highly prevalent, particularly in hospital‐acquired and strong biofilm‐producing isolates. Capsular and regulatory genes (magA) was more frequent in putative hypervirulent and community‐acquired strains, while siderophore genes entB and iucA were strongly associated with biofilm formation and hospital‐acquired infections. A significantly higher resistance to cephalosporins, along with an increased frequency of multidrug‐resistant phenotypes, was detected in hospital‐acquired, classical, and strong biofilm‐forming isolates. ESBL production was significantly more common in hospital‐acquired and biofilm‐forming isolates. Strong biofilm formation was largely associated with classical *K. pneumoniae* and hospital‐acquired infections, whereas putative hypervirulent strains were primarily weak biofilm producers and community‐acquired. These findings highlight the interplay between virulence determinants, biofilm formation, and antibiotic resistance, emphasizing the need for targeted infection control and treatment strategies in North Lebanon.

## Graphical abstract

– Figure design (single panel)

Central icon:

*K. pneumoniae* cell

- **Left panel – Virulence genes (icons):**
- Adhesion: fimH, mrkD → strong biofilm
- Capsule/regulation: magA, rmpA
- Siderophores: entB, iucA → iron uptake
- **Right panel – Outcomes:**
- Biofilm strength (weak → strong gradient)
- Antibiotic resistance (ESBL, MDR)
- **Bottom split:**
- Hospital‐acquired → strong biofilm + MDR
- Community‐acquired → Putative hypervirulence + weak biofilm
- **Arrows: bidirectional arrows showing interplay between virulence ↔ biofilm ↔ resistance.**

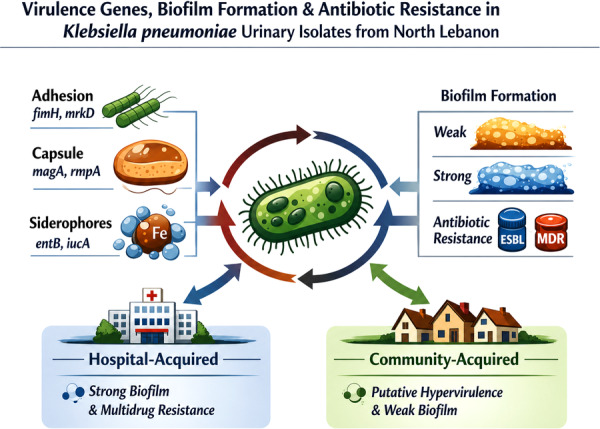

## 1 Introduction

*Klebsiella pneumoniae* (*K. pneumoniae*) is an opportunistic Gram‐negative bacterium of the *Enterobacteriaceae* family and a leading cause of healthcare‐associated infections worldwide. It is responsible for a wide range of clinical syndromes, including urinary tract infections (UTIs), pneumonia, bloodstream infections, and wound infections, with UTIs being among the most prevalent (Ashurst and Dawson 2025). These infections are associated with prolonged hospitalization, increased healthcare costs, and significant morbidity, underscoring the global clinical importance of *K. pneumoniae* (Itani et al. 2024).

The increasing prevalence of antimicrobial‐resistant (AMR) *K. pneumoniae* represents a major public health concern. The pathogen has acquired multiple resistance determinants, particularly against β‐lactams, fluoroquinolones, and aminoglycosides, which has severely restricted treatment options (Ferreira et al. 2019). The global rise of carbapenem‐resistant *K. pneumoniae* (CRKP) is especially alarming, as these strains are often resistant to last‐line therapies and are linked to elevated mortality rates (Karampatakis et al. 2023; Prestinaci et al. 2015).

Pathogenesis of *K. pneumoniae* is multifactorial and relies on a diverse repertoire of virulence determinants. Capsular polysaccharides provide resistance against phagocytosis, fimbriae mediate adhesion and colonization of uroepithelial cells, and siderophores enable iron acquisition within the host environment (Chen et al. 2024; Riwu et al. 2022). Additionally, the hypermucoviscosity phenotype, regulated by genes such as rmpA, enhances invasiveness. Another critical feature is biofilm formation, which facilitates bacterial persistence on urinary tract epithelium and indwelling devices. Biofilms provide a protective niche that limits antibiotic penetration and shields bacteria from host immune responses, contributing to recurrent and chronic infections (Guerra et al. 2022).

Despite extensive global research, regional data remain crucial given the heterogeneity of resistance mechanisms and virulence profiles across geographic settings. In Lebanon, UTIs caused by *K. pneumoniae* constitute a growing health burden, aggravated by widespread antibiotic misuse, inadequate infection control, and the circulation of multidrug‐resistant organisms (Itani et al. 2024). However, comprehensive molecular data on virulence factors, biofilm formation, and resistance genes among urinary *K. pneumoniae* isolates in the northern region are lacking.

This study investigated urinary *K. pneumoniae* isolates obtained from patients in North Lebanon to characterize their antimicrobial resistance patterns, biofilm‐forming ability, and distribution of major virulence determinants. Furthermore, correlations between virulence factors, resistance profiles, and biofilm formation were examined to provide novel insights into the pathogenicity of *K. pneumoniae*. Findings from this study are expected to contribute to a better understanding of local epidemiology and to inform evidence‐based strategies for infection control and therapeutic management.

## 2 Materials and Methods

### 2.1 Sample Collection

This cross‐sectional study included non‐duplicated *K. pneumoniae* isolates recovered from urine specimens of patients with urinary tract infections (UTIs) in North Lebanon. The isolates were collected from two hospitals—Monla Hospital and Dar Al‐Chifaa Hospital—and from one private laboratory (Kadisha Laboratory) between September 2020 and September 2023. Only isolates from Lebanese patients were included. Hospital‐acquired (HA) infections were defined as those appearing ≥ 48 h after admission or within 30 days of hospitalization; all others were classified as community‐acquired (CA). Isolates were cultured on MacConkey agar and incubated at 37°C for 24–48 h. The isolates included in the study were identified using biochemical gallery tests (API 20E; BioMérieux, Marcy‐l′Étoile, France) or by matrix‐assisted laser desorption/ionization time‐of‐flight mass spectrometry (MALDI‐TOF MS) using a VITEK MS instrument (bioMérieux, Marcy l′Etoile, France).

### 2.2 Antimicrobial Susceptibility Testing

Antimicrobial susceptibility testing was performed using the Kirby‐Bauer disk diffusion method. All laboratory procedures followed the European Committee on Antimicrobial Susceptibility Testing (EUCAST) guidelines for microbiological methods and interpretive criteria (EUCAST: Breakpoint Tables, Version 14.0, 2023). The antibiotics tested included penicillins: amoxicillin (AMX), amoxicillin/clavulanic acid (AMC), piperacillin (PIP), and piperacillin/tazobactam (PPT); cephalosporins: cefalotin (CEF), cefuroxime (CXM), cefoxitin (FOX), ceftazidime (CAZ), and cefepime (CFM); carbapenems: ertapenem (ETP) and imipenem (IMP); quinolones: nalidixic acid (NAL) and ciprofloxacin (CIP); sulfonamides: trimethoprim/sulfamethoxazole (SXT); and aminoglycosides: amikacin (AKM) and gentamicin (GM). All isolates were tested against this antibiotic panel to determine susceptibility patterns and classify resistance phenotypes.

### 2.3 Hypermucoviscosirty Phenotype

The string test was performed on colonies grown overnight on 5% sheep blood agar at 37°C. A positive result was defined as the formation of a viscous string > 5 mm (Kim et al. 2017).

### 2.4 Detection of Virulence and Antimicrobial Resistance Genes

Genomic DNA was extracted from all *K. pneumoniae* isolates using the NucleoSpin® Tissue Kit (MACHEREY‐NAGEL, Düren, Germany), following the manufacturer's protocol. DNA extracts were stored at –20°C until further use. Polymerase chain reaction (PCR) assays were performed to detect key virulence‐associated genes—fimH, mrkD, magA, rmpA, entB, kfu, iucA, and iroN. Phenotypically ESBL‐positive *K. pneumoniae* isolates were subjected to PCR screening for β‐lactamase–encoding resistance genes, including blaTEM, blaSHV, and blaCTX‐M. Each PCR reaction was carried out using gene‐specific primers (Table 1), previously validated in the literature. Amplification conditions were optimized for each gene family, and PCR products were visualized by agarose gel electrophoresis to confirm the presence or absence of target amplicons. Isolates were classified as putative hypervirulent *K. pneumoniae* based on a positive string test together with the detection of at least one hypervirulence‐associated gene, including *rmpA*, *magA*, or *iucA*. Isolates not fulfilling these criteria were classified as classical *K. pneumoniae* (Duarte‐Zambrano et al. 2026; Chou et al. 2016; Neumann et al. 2023a; Shafaie et al. 2025a).

**Table 1:** PCR primers used for detection of virulence and antimicrobial resistance genes in *K. pneumoniae*.

Target genes	Primer sequence (5′–3′)	Amplicon size (bp)	Reference
mrkD	F: CCACCAACTATTCCCTCGAA R: ATGGAACCCACATCGACATT	226	Dan et al. (2023)
fimH	F: TGCTGCTGGGCTGGTCGATG R: GGGAGGGTGACGGTGACATC	550	Dan et al. (2023)
magA	F: GGTGCTCTTTACATCATTGC R: GCAATGGCCATTTGCGTTAG	1283	Kot et al. (2023)
rmpA	F: ACTGGGCTACCTCTGCTTCA R: CTTGCATGAGCCATCTTTCA	516	Kot et al. (2023)
entB	F: ATTTCCTCAACTTCTGGGGC R: AGCATCGGTGGCGGTGGTCA	371	Kot et al. (2023)
kfu	F: ATAGTAGGCGAGCACCGAGA R: AGAACCTTCCTCGCTGAACA	520	Kot et al. (2023)
iucA	F: GCATAGGCGGATACGAACAT R: CACAGGGCAATTGCTTACCT	641	Taha et al. (2024)
iroN	F: CTGTCGGCATCGGTTTTATT R: TGGCGTGTCGATTATTACCA	556	Kot et al. (2023)
blaCTX‐M	F: ATGTGCAGYACCAGTAARGT R: TGGGTRAARTARGTSACCAGA	593	Christophy et al. (2017)
blaTEM	F: ATGAGTATTCAACATTTCCG R: CTGACAGTTACCAATGCTTA	867	Christophy et al. (2017)
blaSHV	F: GGTTATGCGTTATATTCGCC R: TTAGCGTTGCCAGTGCTC	867	Christophy et al. (2017)

### 2.5 Biofilm Formation Assay

Biofilm production was quantified by the microtiter plate method (Zheng et al. 2018). Briefly, standardized bacterial suspensions (1 McFarland) were inoculated in 12‐well polystyrene plates with BHI broth and incubated at 37°C for 24 h. Wells were washed, fixed with methanol, stained with 1% crystal violet, and the bound dye solubilized with 33% glacial acetic acid. Absorbance was measured at 595 nm. Each strain was tested in quadruplicate. Based on the cut‐off OD (ODc = 0.05) (Vuotto et al. 2017), isolates were classified as non/weak (ODm < 2ODc), moderate (2ODc < ODm ≤ 4ODc), or strong (ODm > 4ODc) biofilm producers.

### 2.6 Statistical Analysis

Data were analyzed using SPSS version 25. Associations between virulence genes, resistance patterns, and biofilm production were evaluated using Chi‐square or Fisher's exact tests. Multivariable logistic regression analysis was performed to identify factors independently associated with multidrug resistance (MDR). MDR status was used as the dependent variable, while hospital‐acquired infection, putative hypervirulent phenotype, and biofilm formation were included as independent variables. Adjusted odds ratios (ORs) with 95% confidence intervals (CIs) were reported. A *p*‐value < 0.05 was considered statistically significant.

## 3 Results

Among the 153 *K. pneumoniae* isolates analyzed, significant differences were observed in the distribution of virulence‐associated genes based on the origin of infection (hospital‐acquired vs. community‐acquired), biofilm production capability, and strain type (classical vs. putative hypervirulent). Overall, string test positivity was observed in 103/153 (67.3%) *K. pneumoniae* isolates, indicating a high prevalence of the hypermucoviscous phenotype among the studied isolates. The adhesion‐related genes *mrkD* and *fimH* were highly prevalent overall, with significantly higher frequencies in hospital‐acquired isolates compared to community‐acquired ones (*mrkD*: 92.9% vs. 75.9%, *p* = 0.005; *fimH*: 95.7% vs. 74.7%, *p* < 0.001). These genes were also significantly more common among biofilm‐producing isolates, showing 100% prevalence in strong biofilm producers versus 64.9% and 57.9% in weak producers (*p* < 0.001).

The capsular gene *magA* was more frequently detected in putative hypervirulent and community‐acquired strains. Specifically, *magA* was identified in 28.9% of community‐acquired isolates compared to 10% of hospital‐acquired isolates (*p* = 0.004). The *rmpA* gene showed a strong association with biofilm formation, being present in 59.4% of strong biofilm producers versus 3.5% of weak producers (*p* < 0.001).

The siderophore gene *entB* was universally present in biofilm‐producing isolates (100%) and was significantly more prevalent in hospital‐acquired isolates compared to community‐acquired ones (90% vs. 42.2%, *p* < 0.001). Similarly, the *iucA* gene showed higher prevalence in hospital‐acquired (95.7%) and strong biofilm‐producing strains (100%) compared to their counterparts (*p* < 0.001). No significant differences were observed in the distribution of *kfu* and *iroN* genes across the groups (Table 2).

**Table 2:** Distribution of virulence genes and their association with biofilm formation in hospital‐ and community‐acquired urinary *K. pneumoniae*.

	Hospital‐acquired infections *n* = 70	Community‐acquired infections *n* = 83	*p*‐value	Biofilm producers		Non‐biofilm producers	*p*‐value	Classical *K*. *pneumoniae n* = 50	Putative Hypervirulent *K*. *pneumonia n *= 103	*p*‐value	Total *n* = 153
				Strong biofilm producers *n* = 32	Moderate biofilm producers *n* = 64	Weak biofilm producers *n* = 57					
mrkD	65 (92.86)	63 (75.90)	0.005	32 (100)	59 (92.19)	37 (64.91)	< 0.001	46 (92)	82 (79.61)	0.063	128 (83.66)
fimH	67 (95.71)	62 (74.70)	< 0.001	32 (100)	64 (100)	33 (57.89)	< 0.001	46 (92)	83 (80.58)	0.096	129 (84.31)
magA	7 (10)	24 (28.92)	0.004	1 (3.13)	13 (20.31)	17 (29.82)	0.011	0 (0)	31 (30.10)	< 0.001	31 (20.26)
rmpA	18 (25.71)	5 (6.02)	0.001	19 (59.38)	2 (3.13)	2 (3.51)	< 0.001	17 (34)	6 (5.83)	< 0.001	23 (15.03)
entB	63 (90)	35 (42.17)	< 0.001	32 (100)	64 (100)	2 (3.51)	< 0.001	41 (34)	57 (55.34)	0.001	98 (64.05)
kfu	10 (14.29)	7 (8.43)	0.251	5 (15.63)	6 (9.38)	6 (10.53)	0.646	6 (12)	11 (10.68)	0.808	17 (11.11)
iucA	67 (95.71)	59 (71.08)	< 0.001	32 (100)	64 (100)	30 (52.63)	< 0.001	46 (92)	80 (77.67)	0.029	126 (82.35)
iroN	3 (4.29)	5 (6.02)	0.634	2 (6.25)	3 (4.69)	3 (5.26)	0.949	3 (6)	5 (4.85)	0.765	8 (5.23)

### 3.1 Antimicrobial Resistance Patterns

Table 3 showed that all isolates in the study were completely resistant to amoxicillin and piperacillin, with every strain showing non‐susceptibility to both medications (100% resistance). Resistance to amoxicillin combined with clavulanic acid appeared in 51.4% of hospital‐acquired isolates and 43.4% of community‐acquired isolates, and this difference did not reach statistical significance (*p* = 0.320). More noticeable differences were detected among the cephalosporin group. Resistance to ceftriaxone and cefuroxime was significantly higher in isolates obtained from hospital settings (52.9%) compared with those originating from the community (31.3%), showing a meaningful statistical difference (*p* = 0.007).

**Table 3:** Antimicrobial resistance patterns of *K. pneumoniae* isolates according to infection source, biofilm production, and strain Type.

	Hospital‐acquired infections *n* = 70	Community‐acquired infections *n* = 83	*p*‐value	Biofilm producers		Non‐biofilm producers	*p*‐value	Classical *K*. *pneumoniae n* = 50	Putative Hypervirulent *K*. *pneumoniae n* = 103	*p*‐value	Overall resistance *n* (%) Total = 153
				Strong biofilm producers *n* = 32 (%)	Moderate biofilm producers *n* = 64(%)	weak biofilm producers *n* = 57 (%)					
AMX	70 (100)	83 (100)	—	32 (100)	64 (100)	57 (100)	—	50 (100)	103 (100)	—	153 (100)
AMC	36 (51.43)	36 (43.37)	0.320	24 (75)	26 (40.62)	22 (38.59)	0.002	37 (74)	35 (33.98)	< 0.001	72 (47.1)
PIP	70 (100)	83 (100)	—	32 (100)	64 (100)	57 (100)	—	50 (100)	103 (100)	—	153 (100)
PPT	26 (37.14)	31 (37.35)	0.979	22 (68.75)	17 (26.56)	18 (31.57)	< 0.001	26 (52)	31 (30)	0.009	57 (37.2)
CEF	37 (52.86)	26 (31.33)	0.007	25 (78.12)	24 (37.5)	14 (24.56)	< 0.001	38 (76)	25 (24.27)	< 0.001	63 (41.17)
CXM	37 (52.86)	26 (31.33)	0.007	25 (78.12)	24 (37.5)	14 (24.56)	< 0.001	38 (76)	25 (24.27)	< 0.001	63 (41.17)
FOX	2 (2.86)	7 (8.43)	0.144	1 (3.12)	2 (3.12)	6 (10.52)	0.170	4 (8)	5 (4.85)	0.438	9 (5.88)
*CAZ*	33 (47.14)	26 (31.33)	0.045	22 (68.75)	24 (37.5)	13 (22.80)	< 0.001	34 (68)	25 (24.27)	< 0.001	59 (38.56)
*CFM*	33 (47.14)	22 (26.51)	0.088	22 (68.75)	22 (37.5)	11 (19.29)	< 0.001	34 (68)	21 (20.38)	< 0.001	55 (35.94)
ETP	2 (2.86)	2 (2.41)	0.863	1 (3.12)	1 (1.56)	2 (3.50)	0.783	3 (6)	1 (0.97)	0.067	4 (2.61)
IMP	2 (2.86)	1 (1.20)	0.463	1 (3.12)	1 (1.56)	1 (3.50)	0.865	3 (6)	0 (0)	0.033	3 (1.96)
*GM*	17 (24.29)	16 (19.28)	0.456	12 (37.5)	11 (17.18)	10 (17.54)	0.048	17 (34)	16 (15.53)	0.009	33 (21.56)
*AKN*	2 (2.86)	3 (3.61)	0.793	1 (3.12)	2 (3.12)	2 (3.50)	0.992	2 (4)	3 (2.91)	0.723	5 (3.26)
NAL	19 (27.14)	19 (22.89)	0.546	12 (37.5)	14 (21.87)	12 (21.05)	0.175	18 (36)	20 (19.41)	0.030	38 (24.83)
*CIP*	17 (24.29)	16 (19.28)	0.453	11 (34.37)	11 (17.18)	11 (19.29)	0.135	16 (32)	17 (16.50)	0.037	33 (21.56)
*SXT*	34 (48.57)	30 (36.14)	0.870	20 (62.5)	24 (37.5)	20 (35.08)	0.028	27 (54)	37 (35.92)	0.033	64 (41.83)
MDR	40 (57.14)	27 (32.53)	0.004	26 (81.25)	25 (39.06)	16 (28.07)	< 0.001	39 (78)	28 (27.18)	< 0.001	67 (43.79)
ESBL	35 (50)	20 (24.10)	0.001	24 (75)	22 (34.37)	9 (15.78)	< 0.001	34 (68)	21 (20.38)	< 0.001	55 (35.94)

A similar pattern was observed with ceftazidime, where hospital‐acquired isolates demonstrated a resistance rate of 47.1%, while community‐acquired strains showed a rate of 31.3%. This difference was significant (*p* = 0.045).

Biofilm formation strongly influenced antibiotic resistance. Strains classified as strong biofilm producers exhibited markedly higher resistance to several drugs when compared with moderate and weak biofilm producers. Resistance among strong biofilm formers reached 75% for amoxicillin–clavulanic acid, 78.1% for ceftriaxone, 68.8% for ceftazidime, and 68.8% for cefixime. All of these comparisons showed highly significant differences (*p* < 0.001).

The distribution of multidrug‐resistant strains further reflected this trend. Hospital‐acquired isolates demonstrated a significantly higher rate of MDR (57.1%) compared with isolates obtained from community settings (32.5%), with a strong statistical association (*p* = 0.004). Among strains capable of forming strong biofilms, MDR reached 81.3%, while only 28.1% of non‐biofilm or weak biofilm producers showed the same pattern. This difference was highly significant (*p* < 0.001).

A similar distribution was observed for extended‐spectrum beta‐lactamase (ESBL) activity. ESBL production was present in 50% of hospital‐acquired isolates and in 24.1% of community‐acquired isolates, demonstrating a statistically significant difference (*p* = 0.001). Strong biofilm producers again showed much higher ESBL activity (75%) compared with weak producers (15.8%), also highly significant (*p* < 0.001). No statistically meaningful differences were reported for imipenem, amikacin, or cefoxitin, as resistance to these antibiotics was similar across the groups studied.

When comparing classical *K. pneumoniae* strains with the putative hypervirulent form, the classical strains exhibited significantly greater resistance to amoxicillin–clavulanic acid, ceftriaxone, ceftazidime, and a higher overall burden of MDR, with all comparisons showing strong statistical significance (*p* < 0.001).

PCR screening for β‐lactamase–encoding genes revealed a frequent detection of resistance determinants among phenotypically ESBL‐positive *Klebsiella pneumoniae* isolates. The *blaTEM* gene was detected in 50 isolates (90.9%), while *blaCTX‐M* was present in 42 isolates (76.4%). In comparison, the *blaSHV* gene was identified in 32 isolates (58.2%).

### 3.2 Biofilm Production Characteristics

Biofilm production varied significantly between hospital‐acquired and community‐acquired infections. Strong biofilm producers were predominantly associated with hospital‐acquired infections (90.6%) compared to community‐acquired infections (9.4%, *p* < 0.001). In contrast, weak biofilm producers were more frequently observed in community‐acquired infections (85.9%) compared to hospital‐acquired infections (14.0%, *p* < 0.001). Moderate biofilm producers were more evenly distributed but still significantly more prevalent in community‐acquired cases (48.4%) compared to hospital‐acquired ones (51.6%, *p* < 0.001) (Table 4).

**Table 4:** Distribution of clinical origin and strain type of *K. pneumoniae* according to biofilm production levels.

	Biofilm producers		Non‐biofilm producers	*p*‐value	Total
	Strong biofilm producers *n* = 32 (%)	Moderate biofilm producers *n* = 64(%)	weak biofilm producers *n* = 57 (%)		
Community‐acquired infections	3 (9.37)	31 (48.43)	49 (85.96)	< 0.001	83
Putative Hypervirulent *K. pneumoniae*	8 (25)	48 (75)	47 (82.45)	< 0.001	103

Regarding strain type, classical *K. pneumoniae* strains exhibited a higher prevalence of strong biofilm formation (75%) compared to putative hypervirulent strains (25%, *p* < 0.001), whereas putative hypervirulent strains were predominantly weak biofilm producers (82.5%) compared to classical strains (17.5%, *p* < 0.001) (Table 4).

### 3.3 Strain Type and Infection Origin

Classical *K. pneumoniae* strains were predominantly associated with hospital‐acquired infections, comprising 84% (42/50) of isolates in this group, compared to only 16% in community‐acquired infections (*p* < 0.001). Conversely, putative hypervirulent strains were more commonly isolated from community‐acquired infections, representing 72.8% (75/103) of cases in this group, versus 27.2% from hospital‐acquired infections (*p* < 0.001).

Overall, hospital‐acquired infections accounted for 45.8% of the total isolates, with a predominance of classical strains, while community‐acquired infections constituted 54.2%, largely driven by putative hypervirulent strains. These findings underscore a significant association between infection origin, strain type, virulence gene distribution, biofilm production capacity, and antimicrobial resistance (Table 5).

**Table 5:** Distribution of hospital‐ and community‐acquired infections among classical and putative hypervirulent *K. pneumoniae* isolates.

	Classical *K*. *pneumoniae n* = 50	Putative Hypervirulent *K*. *pneumoniae n* = 103	*p*‐value	Total *n* = 153
Hospital‐acquired infections *n* = 70	42 (84)	28 (27.18)	< 0.001	70 (45.75)
Community‐acquired infections *n* = 83	8 (16)	75 (72.81)	< 0.001	83 (54.24)

### 3.4 Independent Predictors of MDR Among *Klebsiella pneumoniae* Isolates

Multivariable logistic regression analysis was conducted to identify independent predictors of MDR among *K. pneumoniae* isolates. After adjustment, putative hypervirulent phenotype was significantly associated with MDR, with putative hypervirulent isolates showing higher odds of MDR compared with classical isolates (adjusted OR = 8.485, 95% CI: 3.180–22.638, *p* < 0.001). Biofilm formation was also independently associated with MDR (adjusted OR = 7.428, 95% CI: 2.047–26.961, *p* = 0.002). However, hospital‐acquired infection was not significantly associated with MDR after adjustment (adjusted OR = 2.028, 95% CI: 0.748–5.498, *p* = 0.165). These findings suggest that putative hypervirulence and biofilm formation may contribute to the MDR profile of the studied *K. pneumoniae* isolates (Table 6).

**Table 6:** Multivariable logistic regression analysis of factors associated with MDR among *K. pneumoniae* isolates.

Independent variable	*p*‐value	Adjusted OR, Exp(B)	95% CI
Hospital‐acquired infection	0.165	2.028	0.748–5.498
Putative hypervirulent	< 0.001	8.485	3.180–22.638
Biofilm formation	0.002	7.428	2.047–26.961

## 4 Discussion

*K. pneumoniae* of two different strains, classical (cKP) and hypervirulent (hvKP), causing both community‐acquired and hospital‐acquired infections has become a serious threat to public health in recent years. High resistance in *K. pneumoniae* was established due to extensive use of antibiotics in treating this infection (Kot et al. 2023). In this study, significant differences were assessed based on the distribution of virulence‐associated genes according to the origin of infection, biofilm production capability, and strain type. Additionally, the resistance of the tested isolates to a variety of antibiotics was evaluated. The interaction between antimicrobial resistance, virulence‐associated genes, and biofilm formation in *K. pneumoniae* remains poorly understood; therefore, the combined evaluation of these factors is important. In line with this, Mirzaie and Ranjbar reported the coexistence of antibiotic resistance and virulence‐associated genes among clinical *K. pneumoniae* isolates, supporting the relevance of investigating resistance and virulence determinants together (Mirzaie and Ranjbar 2021).

MrkD determines the properties of adhesion and plays a role in determining the binding specificity of the fimbriae (Fang et al. 2021). Similarly, *fimH* gene expression, encoding FimH, has a vital role in strong biofilm formation by promoting bacteria's binding to surfaces (Li and Ni 2023). Overall, the adhesion‐related genes *mrkD* and *fimH* were highly prevalent in our study. Significantly higher levels of these virulent genes were detected in hospital‐acquired isolated compared to community acquired isolated (*mrkD*: 92.9% vs 75.9%; *fimH*: 95.7% vs. 74.7%). In a similar study, *fimH* gene was prevalent in 91.7% and *mrkD* gene showed a frequency of 96% of *K. pneumoniae* isolates from hospitalized patients in Poland (Kot et al. 2023). In our study, adhesion‐related genes were more prevalent among biofilm‐producing isolates, with both *mrkD* and *fimH* detected in 100% of strong biofilm producers compared with 64.7% and 57.9% of weak producers, respectively. This supports previous findings linking fimbrial adhesion genes, such as *mrk* and *fim* genes, to enhanced biofilm formation in *K. pneumoniae* isolates (Shafaie et al. 2025b). Additionally, according to results reported by Makhrmash et al. the PCR method associated the presence of *fimH, mrkD* and *mrkA* genes (87.5%, 53.6% and 46.4% respectively) to biofilm formation in different *K. pneumoniae* isolates (Makhrmash et al. 2022). In summary, our study confirms the contribution of adhesion‐related genes to hospital acquired infection caused by *K. pneumoniae* and its ability to form strong biofilms.

The presence of capsular polysaccharide (CPS), which is encoded and regulated by several genes including *rmpA* and *magA* contributes to the pathogenesis of *K. pneumoniae (*Xue et al. 2025 *). magA* gene is known to have a correlation with the hyper‐production of K1 serotype capsule (Ventura et al. 2022). In our study, *magA* was detected only among putative hypervirulent *K. pneumoniae* isolates, with a prevalence of 30.1%, while none of the classical isolates carried this gene. This finding is consistent with previous reports linking *magA* to the hypermucoviscous phenotype of *K. pneumoniae* (Ventura et al. 2022). Another study conducted in West Bengal, India, stated that hvKP was more commonly linked with K1, K2, K5, K20, K54, and K57 than cKP (Mukherjee et al. 2023). In a study in Poland, 19.2% of isolates from hospitalized patients detected the presence of the K1 capsule serotype encoded by chromosomal mucoviscosity‐associated gene A ‐ *magA* (Kot et al. 2023). All in all, these results provide a reliant proof that *magA* gene plays a role in the mucoviscosity trait of hypervirulent *K. pneumoniae*.

In our study, *rmpA* was strongly associated with biofilm formation, being detected in 59.4% of strong biofilm producers compared with 3.5% of weak producers (*p* < 0.001). This finding supports previous reports linking *rmpA* and other virulence‐associated genes to enhanced biofilm formation in *K. pneumoniae*. In an Iranian study, PCR analysis showed that isolates with strong or moderate biofilm‐forming ability were positive for *mrkA*, *mrkD*, and *rmpA* genes (Bakhtiari et al. 2021). However, in a study in Spain, biofilm‐forming strain were seen to lack *rmpA* gene (*p* < 0.001) (Ballén et al. 2021). This discrepancy may be explained by differences in strain characteristics, study design, biofilm assay conditions, or geographic variation. Overall, our findings support the potential contribution of *rmpA* to the biofilm‐associated virulence profile of *K. pneumoniae*.

The *entB* gene plays a role in iron transport in gram negative bacteria and is highly seen in *K. pneumoniae (*Dan et al. 2023). The *iucA* gene also plays a role in the formation of the siderophore aerobactin (Ballén et al. 2021). According to our results, siderophore genes *entB and iucA* were significantly more prevalent in hospital‐acquired isolates compared to community acquired ones (entB: 90% and 42.2% respectively; *p* < 0.001; iucA: 95.71% and 71.08% respectively; *p* < 0.001). Similarly, *entB* and *iucA* were highly prevalent among biofilm‐producing isolates, being detected in 100% of strong and moderate biofilm producers, while their prevalence was lower among weak producers. This is consistent with previous reports linking siderophore‐related genes, including *entB* and *iucA*, with the biofilm‐forming ability and virulence profile of *K. pneumoniae* (Allami et al. 2024). As a result, iron sequestering systems, by harvesting iron, are virulence factors that enhance the pathogenicity of *K. pneumoniae* by promoting its ability to form biofilms.

In this study, MDR was significantly associated with infection source, biofilm formation, and strain category. MDR was more frequent among hospital‐acquired isolates than community‐acquired isolates (57.1% vs. 32.5%; *p* = 0.004), among strong biofilm producers compared with weak producers (81.3% vs. 28.1%; *p* < 0.001), and among classical *K. pneumoniae* isolates compared with putative hypervirulent isolates (78.0% vs. 27.2%; *p* < 0.001). In a Chinese study by L. Li et al. results were consistent with ours regarding biofilm formation profile, where biofilm formation in MDR isolates showed significantly higher proportion than in non‐MDR isolates with a p value *p* < 0.05 (Li et al. 2024). However, a study reported that MDR *K. pneumoniae* infections are being increasingly reported in CA infections and restricting antimicrobial resistance to hospitals are no longer applicable as their results indicated the high prevalence of MDR *K. pneumoniae* in CA infections in Brazil (Monteiro et al. 2025). Our results regarding strain type and MDR was also inconsistent with results reporting high multidrug‐resistant hvKP in China (Hetta et al. 2025). hvKP is acquiring MDR resistance genes through two ways: type I hvKP where resistance genes or plasmids are being transferred through horizontal gene transfer, and type II hvKP are emerging by the transfer of p‐LVPK‐like virulence plasmid into classical MDR *K. pneumoniae* (Hetta et al. 2025). Similarly, Huang et al. reported that despite the fact that hypervirulent strains were historically reported as antibiotics susceptible, the wide use of antibiotics and extensive environmental pressures are allowing MDR strains to acquire hypervirulent characteristics and emerge as MDR hvKP (Huang et al. 2025). Although our results align with earlier results regarding high incidence of MDR classical *K. pneumoniae*, the emergence of MDR‐hvKP is being highly reported and it possess a global health issue raising concerns worldwide.

ESBL production showed a similar pattern, being more frequent among strong biofilm producers than weak producers (75.0% vs. 15.8%) and among hospital‐acquired isolates compared with community‐acquired isolates (50.0% vs. 24.1%). This supports previous findings suggesting a relationship between ESBL production and biofilm‐forming capacity in *K. pneumoniae* (Gao et al. 2024). However, in Portugal, Sabença et al. reported no correlation between strong biofilm production profile and ESBL in *K. pneumoniae* isolates (Sabença et al. 2023). Therefore, results are contradicting in this manner. This could be a result of different resistance patterns in different geographical locations.

In the present study, PCR screening of β‐lactamase–encoding genes demonstrated a high prevalence of extended‐spectrum β‐lactamase (ESBL) determinants among *K. pneumoniae* isolates. The *blaTEM* gene was the most frequently detected resistance gene (90.9%), followed by *blaCTX‐M* (76.4%) and *blaSHV* (58.2%). Our findings partially contrast with several studies conducted in Lebanon, where *blaCTX‐M* has consistently been reported as the most prevalent ESBL gene among *Enterobacterales*, including *K. pneumoniae* (Kanj et al. 2008). This discrepancy may reflect differences in sample sources, hospital settings, antibiotic‐prescribing practices, and temporal variations in circulating resistance clones. Nevertheless, the high prevalence of *blaCTX‐M* observed in our isolates confirms its continued dominance as a key driver of ESBL‐mediated resistance in the region. In neighboring countries, variability in ESBL gene distribution has also been documented. For example, studies from Iraq reported *blaTEM* as the most prevalent ESBL gene, followed by *blaCTX‐M*, which is in line with the dominance of *blaTEM* observed in our study (Ahmad Hamad and Khadija 2019).

The frequent coexistence of multiple ESBL genes within the same isolate, as observed in previous studies, further supports our findings. A study done in north Lebanon reported that 88% of ESBL‐producing isolates harbored multiple resistance genes, with *blaCTX‐M* (92%) being the most predominant, followed by *blaTEM* (86%), *blaSHV* (64%), and *blaOXA* (28%) (Hamwi and Salem‐Sokhn 2023). The presence of multiple β‐lactamase genes may enhance resistance levels and facilitate the persistence and spread of multidrug‐resistant strains through plasmid‐mediated horizontal gene transfer.

Similarly, Saria A. El‐Hariri et al. demonstrated that ESBL genes were detected in both *Escherichia coli* and *K. pneumoniae*, with *blaTEM* being the most prevalent gene, followed by *blaCTX‐M* and *blaSHV* (El‐Hariri et al. 2023). These findings align with our results and reinforce the notion that *blaTEM* remains a significant contributor to ESBL production, despite the global expansion of *blaCTX‐M* enzymes. Overall, the frequent detection of ESBL‐encoding genes among ESBL‐positive *K. pneumoniae* isolates highlights the need for continued molecular surveillance, antimicrobial stewardship, and infection‐control measures to limit the spread of resistant strains and guide appropriate empirical therapy.

Bacteria can adhere to living and non‐living surfaces by the production of biofilms. Biofilms are composed of microbial aggregates in a matrix composed of CPS, DNA, and proteins (Dan et al. 2023). Biofilm production varied significantly between hospital‐acquired and community‐acquired infections. Strong biofilm producers were predominantly associated with hospital‐acquired infections (90.6%) compared to community‐acquired infections (9.4%, *p* < 0.001). However, weak biofilm production showed an association with community acquired infections were 49 (85.96%) of the isolates were weak producers. In a study done by Di Domenico et al. in Rome, Italy on carbapenem resistant klebsiella pneumoniae (CRKP), an outstanding cause of nosocomial infections, 55.8% of isolates were strong biofilm producers while 44.2% were weak producers (Di Domenico et al. 2020). Another study results found that among hospital acquired infections, the highest prevalence of strong biofilm producers was among *K. pneumoniae* with a 50.3% prevalence and less that 20% were weak producers (Ben‐Amram et al. 2024). This can be explained by the ability of *K. pneumoniae* to colonize medical devices (such as catheters, nasogastric tubes, and ventilators) leading to higher resistance episodes to antibiotics and serious difficult‐to‐treat nosocomial infections.

Regarding strain type, classical *K. pneumoniae* strains exhibited a higher prevalence of strong biofilm formation (75%) compared to hypervirulent strains (25%, *p* < 0.001), whereas hypervirulent strains were predominantly weak biofilm producers (82.5%) compared to classical strains (17.5%, *p* < 0.001). In a study conducted in Germany, the results aligned with our results where it was stated that hvKP isolates showed low ability of biofilm formation (Neumann et al. 2023b). Similar results were reported by a research study in China, where cKP had more capabilities than hvKP of biofilm formation (0.6686 ± 0.0661 vs. 0.2625 ± 0.0579, *p* = 0.033) (Li et al. 2022). However, a study on hvKP causing UTI in kidney stone patients, hvKP isolates showed higher biofilm forming activity than cKP (94.4% vs. 86%) which is inconsistent with our results (Zafar et al. 2019). This may be explained by the specific site of infection (Urinary tract) studied by Zafar et. al, where biofilm formation can be affected by the site. Biofilm formation may have also been differed due to different growth requirements provided by different studies or even by variations in strains studied which show variations in their genetics.

In our study, the two strains investigated in the studied *K. pneumoniae* isolates showed a surprising significant association with the source of acquisition of infection. Classical *K. pneumoniae* showed a predominant association with hospital‐acquired infections comprising 84% of isolates in this group compared to 16% in community‐acquired infections while hvKP showed the opposite results (predominated in community acquired infections). However, surprisingly, a study in Beijing showed that hvKP may be emerging to replace cKP as the predominant strain of *K. pneumoniae* in nosocomial infections (Liu et al. 2020). In a meta‐analysis study in India, results showed that CA‐hvKP prevalence is still higher than HA‐hvKP; however, the prevalence of HA‐hvKP showed an increase from 25.9% to 47.1% in 2006–2018 and 2019–2024 respectively (Gupta et al. 2025). More importantly, a recent global genomic analysis of 2097 hvKP isolates reported that 41.58% of hvKP isolates were multidrug‐resistant and 33.29% were carbapenem‐resistant, highlighting the worldwide emergence of hypervirulent strains with clinically important resistance profiles (Jiang et al. 2025). The increase in the emergence of hvKP infections in health‐care settings can be accounted to several results and raises several concerns. A suggested explanation states that hvKP strains are acquiring adaptation to nosocomial settings due to genetic mutations in their virulence genes that allow them to increase their adherence and colonization of health‐care devices and environment. This would increase concerns and be problematic as management protocols for hypervirulent strains will definitely differ from those for classical strains and awareness should be raised for a better patient diagnosis and care (Liu et al. 2020).

This study has several strengths, including the simultaneous evaluation of virulence genes, biofilm formation, and antimicrobial resistance, as well as the inclusion of both hospital‐ and community‐acquired isolates, providing a comprehensive picture of *K. pneumoniae* pathogenicity in North Lebanon. However, there are limitations. The sample size was relatively small and limited to three sites, which may reduce generalizability. Only selected virulence and β‐lactamase genes were analyzed, and biofilm formation may vary under different conditions. Additionally, the cross‐sectional design prevents assessment of temporal trends, and clinical outcome data were not included, limiting correlations between bacterial characteristics and patient prognosis. Despite these limitations, the findings provide valuable insights into the local epidemiology of urinary *K. pneumoniae*.

## 5 Conclusion

This study demonstrates that urinary *K. pneumoniae* isolates in North Lebanon exhibit distinct profiles of virulence, biofilm formation, and antimicrobial resistance. Classical strains are strongly associated with hospital‐acquired infections, multidrug resistance, and robust biofilm formation, while putative hypervirulent strains are more prevalent in community‐acquired infections with weaker biofilm capacity. Adhesion, capsular, and siderophore genes play key roles in virulence and persistence, particularly in biofilm‐producing isolates. The strong correlation between biofilm formation and multidrug resistance underscores the challenge in managing *K. pneumoniae* infections. These findings provide critical insights into local epidemiology and emphasize the importance of continuous surveillance, judicious antibiotic use, and tailored infection control measures to limit the spread of highly virulent and resistant *K. pneumoniae* strains.

## Author Contributions

**Mazen Zaylaa:** conceptualization (lead), investigation (lead), formal analysis (lead), writing – review and editing (lead), supervision (lead). **Rayan Farha:** conceptualization (equal), formal analysis (equal), writing – original draft (equal). **Jana Taleb:** formal analysis (equal), writing – original draft (equal). **Amal Saad:** writing – original draft (equal). **Omar El Gharbawy:** formal analysis (equal). **Ricardo Sarraf:** writing – review and editing (equal). **Ibtihal Kassam:** writing – review and editing (equal). **Chadi Hamdach:** writing – review and editing (equal). **Nisrine Bissar:** writing – review and editing (equal).

## Funding

The authors have nothing to report.

## Ethics Statement

All experiments in this study were performed in accordance with the relevant guidelines and regulations. The study was approved by the Institutional Review Board at Beirut Arab University under approval number (2025‐H‐0156‐M‐R‐0710).

## Conflicts of Interest

The authors declare no conflicts of interest.

## Data Availability

The data that support the findings of this study are available on request from the corresponding author. The data are not publicly available due to privacy or ethical restrictions.
